# Automated Detection of Patients at High Risk of Polypharmacy including Anticholinergic and Sedative Medications

**DOI:** 10.3390/ijerph20126178

**Published:** 2023-06-19

**Authors:** Amirali Shirazibeheshti, Alireza Ettefaghian, Farbod Khanizadeh, George Wilson, Tarek Radwan, Cristina Luca

**Affiliations:** 1 AT Medics Ltd., London SW2 4QY, UK; a.shirazibeheshti@nhs.net (A.S.); alireza.e@nhs.net (A.E.); tradwan@nhs.net (T.R.); 2 Operation & Information Management, Aston Business School, Birmingham B4 7UP, UK; khanizaf@aston.ac.uk; 3 School of Computing and Information Science, Anglia Ruskin University, Cambridge CB1 1PT, UK

**Keywords:** cluster analysis, decision making, drug interactions, polypharmacy, risk factors, unsupervised machine learning

## Abstract

Ensuring that medicines are prescribed safely is fundamental to the role of healthcare professionals who need to be vigilant about the risks associated with drugs and their interactions with other medicines (polypharmacy). One aspect of preventative healthcare is to use artificial intelligence to identify patients at risk using big data analytics. This will improve patient outcomes by enabling pre-emptive changes to medication on the identified cohort before symptoms present. This paper presents a mean-shift clustering technique used to identify groups of patients at the highest risk of polypharmacy. A weighted anticholinergic risk score and a weighted drug interaction risk score were calculated for each of 300,000 patient records registered with a major regional UK-based healthcare provider. The two measures were input into the mean-shift clustering algorithm and this grouped patients into clusters reflecting different levels of polypharmaceutical risk. Firstly, the results showed that, for most of the data, the average scores are not correlated and, secondly, the high risk outliers have high scores for one measure but not for both. These suggest that any systematic recognition of high-risk groups should consider both anticholinergic and drug–drug interaction risks to avoid missing high-risk patients. The technique was implemented in a healthcare management system and easily and automatically identifies groups at risk far faster than the manual inspection of patient records. This is much less labour-intensive for healthcare professionals who can focus their assessment only on patients within the high-risk group(s), enabling more timely clinical interventions where necessary.

## 1. Introduction

It is common medical practise for patients to be safely prescribed more than one drug, thus benefiting from the simultaneous treatment of multiple conditions. This practice, especially where this involves more than five medications, is called polypharmacy [1,2,3,4], with extreme polypharmacy referring to ten or more medications [5]. Sometimes, however, polypharmacy can give rise to adverse drug reactions (ADRs) where the effect of one drug is changed in the presence of other drugs, potentially resulting in increased toxicity [6,7,8]. Approximately 10% of consultations in a primary healthcare setting are related to ADRs and 60–70% of serious ADRs are preventable but are often inadvertently overlooked [9,10]. According to [11], in 2021 the estimated cost per year of avoidable drug related problems for the United Kingdom National Health Service (UK-NHS) was £98,462,582, consuming 181,626 bed-days per year.

There are two important factors that may exacerbate the detrimental effects of polypharmacy. Firstly, patients often look for treatment of the associated new symptoms, but any potential link between the symptoms and the medicines they are already taking may go unrecognised by the healthcare professional. Patients may therefore be prescribed new medicines to counter the adverse effects of the drug, which may inadvertently worsen the problems [12]. Secondly, with aging, the risk of developing chronic diseases and thus ADRs related to multiple drug prescription increases [13,14,15,16,17]. Indeed, prolonged anticholinergic and sedative medications are highly correlated with worsening cognition and decline in physical functions among the elderly [18,19,20]. This issue is well known, with the absolute risk of any single anticholinergic medicine described by the Anticholinergic Cognitive Burden (ACB) scale [21] (with a later supplement by [22] and [23]). According to different studies, the prevalence of polypharmacy ranges from 34% to 65% in older patients, resulting in increased hospital admissions [15,24,25]. One approach to mitigate this effect is de-prescription [5,18,26,27], taking into account that older adults with co-morbidities may benefit less from drugs due to the early medical harm prevailing over the later intended positive effects [28].

The safe prescription of medicines is fundamental to the role of the healthcare professional who, in traditional practice, needs to be knowledgeable and vigilant about the risks associated with drugs and their interactions with other medicines at the individual patient level. Research on polypharmacy has focused, in general, on de-prescription. The aim of the current study was to present a method of identifying patients at high risk of polypharmacy using big data analytics according to their medication profile. Whilst drug dose, patient weight, age, and other factors contribute to polypharmaceutical risk, these were not the focus of the current study. The reason for using Artificial Intelligence (AI) techniques is that they can handle large raw data (such as drug databases and patient medication records) analysis. An AI-powered decision support system can assist health professionals in making informed decisions regarding polypharmacy.

This work contributes to scientific knowledge in two ways; firstly, groups of patients at risk from drug–drug polypharmacy and polypharmacy within the anticholinergic medicine group were identified using novel metrics and mean-shift clustering. Secondly, the automated identification of the highest risk cluster(s) represents an efficient and significant reduction in the data necessary for clinical manual appraisal, typically extracting tens from potentially hundreds of thousands of patient records.

In a professional context, the automated, easy, and rapid recognition of patients at high risk of polypharmacy has marked benefits for patient outcomes (more rapid intervention) and for health management businesses (reducing the time-intensive manual data inspection).

This paper is organised as follows. Section 2 describes the dataset and the methodologies used in this research. Section 3 discusses the experiments performed and their outcomes. A comparison with the state-of-the art is presented is Section 4. Finally, the conclusions are drawn in Section 5.

## 2. Materials and Methods

### 2.1. Drug Interaction and Patient Data Sources

The ePACT2 online archive (https://www.nhsbsa.nhs.uk/epact2, accessed on 28 November 2020) allows authorised users access to prescription data held by the UK-NHS prescription services. This archive was consulted as it includes hospital admission data due to adverse drug reactions and so allows the evaluation of which drugs may be most responsible. Some information for the first quarter of 2019 is presented in Table 1. These data were the most recent available at the time the current study commenced, and comparison with data from two randomly selected quarters from the 3 years prior to this did not reveal any significant differences. The table shows that, whilst polypharmacy caused by the interaction between different medicine groups is important, the prescription of multiple anticholinergic medications within that single group is itself an important source of hospital admissions. Risk rate, shown in column 2, represents the number of patients admitted per 10,000 hospital admissions due to the consequences of the prescription of single or multiple drugs. The present study therefore focused on two aspects of polypharmaceutical risk, one based on the interaction between multiple medications of anticholinergic drugs and the other one based on the interaction between different medicine groups (irrespective of whether one group is anticholinergic).

The ACB scale described earlier recognises three classes of anticholinergic risk where medicines in class 1 have the lowest risk and those in class 3 have the highest risk. In addition, the UK British National Formulary—National Institute for Health and Care Excellence (BNF-NICE) website (https://bnf.nice.org.uk/interaction/, accessed on 22 February 2021) uses an Interaction Severity (IS) score between any two medicine groups. The IS also has three risk levels from 1 (lowest) to 3 (highest) but, unlike the ACB, the score is relative—measured against the lowest risk of the two pairings.

### 2.2. Data, Instruments and Pre-Processing

For the current study, access to a data set of 300,000 patient records registered with the largest provider of primary care services to the NHS in England was utilised (AT Medics Ltd, London, UK). The data that support the findings of this study are available from the NHS bulk data repository. Restrictions apply to the availability of these data, which were used under license for the current study. To ensure complete data, the AT Medics’ patient database was parsed to identify only patient records whose prescriptions were active; those with a historical prescription treatment that had ceased were excluded. These active patients were then checked for an entry in [23], and their anticholinergic drug(s) and ACB scores(s) were extracted.

The cumulative effect of taking one or more anticholinergic drugs was measured using a Weighted Anticholinergic Risk Score (WARS) calculated using Equation (1) for each patient.

(1) $$WARS=n_{c1}*S_{c1}+n_{c2}*S_{c2}+n_{c3}*S_{c3},$$

where *n*_*c*1_, *n*_*c*2_, and *n*_*c*3_ refer to the number of anticholinergic drugs prescribed to a patient, which belongs to classes *c*1, *c*2, and *c*3, respectively. *S*_*c*1_, *S*_*c*2_, and *S*_*c*3_ are the related anticholinergic risk scores associated with each class; i.e., *S*_*c*1_ = 1, *S*_*c*2_ = 2, and *S*_*c*3_ = 3.

With regards to one-to-one drug–drug interactions, a similar approach to that for WARS can be taken, where a Weighted Interaction Risk Score (WIRS) per patient can be derived by using Equation (2).

(2) $$WIRS=n_{mi}*S_{mi}+n_{mo}*S_{mo}+n_{se}*S_{se},$$

where *n*_mi_, *n*_mo_, and *n*_se_ refer to the number of drug pairs prescribed to a patient with mild, moderate, and severe interactions, respectively. *S*_mi_ = 1, *S*_mo_ = 2, and *S*_se_ = 3 are the degrees to which an interaction is severe.

Data pre-processing to derive WIRS values is more involved than that for WARS because many prescription medicines are a mixture of drugs or can even have different names for the same drug, so it is necessary for drug references to be standardised and IS values to be combined so as to generate a WIRS score per patient. This WIRS pre-processing was undertaken in three steps. Firstly, as with WARS, the AT Medics’ patient database was parsed to identify those patients currently receiving a prescription. Secondly, for this subset, each patient’s prescribed medicines were compared with those listed in the BNF-NICE database which, if present, lists the IS score for a number of drug–drug pairings. The AT Medics’ database prescription data include medicines and, where applicable, their drug components referenced in a form that matches the BNF-NICE database, allowing standardisation and cross-referencing between the two resources. Thirdly, the drug–drug IS scores for the drugs prescribed to each patient were then combined to derive the WIRS score per patient. This final step was undertaken by generating an interaction matrix per patient. Each element of the matrix records the relevant pairing IS score (as listed in the BNF-NICE database) or defaults to 0; the overall WIRS score for that patient is then half the sum of all the elements in the matrix (to avoid double-counting the paired values). As an example, Table 2 shows the interaction matrix for a random patient that lists seventeen severe interactions between the drugs listed, so the overall WIRS score is 51. Note that there is no direct relation between WARS and WIRS. The WARS score reflects the risk of polypharmacy specifically from anticholergenic medication and is derived from absolute ACB measures, whilst the WIRS score reflects the broader relative risk of all IS one-to-one drug group pairings (which may or may not include an anticholinergic drug group as one of the pairs).

### 2.3. Mean-Shift Clustering Technique for Polypharmaceutical Risk Identification

WIRS and WARS scores per patient were calculated as described in the previous section, excluding those with a risk score of 0, and were grouped into three categories:

(a) WARS—a single vector of 18,568 patients (6.2%) flagged as medicated with one or more anticholinergic drugs (mean age 46.93 ± 22.10);
(b) WIRS—a single vector of 8856 patients (3.0%) flagged as medicated with one or more medicine groups and therefore at interactive risk from polypharmacy (mean age 58.96 ± 17.50);
(c) WARS and WIRS—a double vector of 4318 patients (1.4%, mean age 59.02 ± 17.30), representing the intersection between categories (a) and (b) (i.e., patients with both WIRS and WARS).

All the medication records were extracted from the database in mid-March of 2020.

The risk metric for categories (a) and (b) are each based on a single risk score, either WARS or WIRS, and on viewing these values for a given patient, the healthcare professional would decide which of the two is the most significant. However, it is not clear if a patient with a higher WARS is at greater risk than a patient with a higher WIRS. Whilst both scores could be combined into a single feature in some way, most health professionals prefer to work with established clinically recognised measures so as to have options for a judgement call between different prioritisation strategies. For this reason, the category (c) data do not combine the scores but rather use a two dimensional vector of two elements per patient for the respective WARS and WIRS values.

Clustering is an unsupervised learning method used in this research to group together patients with similar characteristics (here, similar risk scores). There are two algorithmic approaches that can be used. One approach requires defining the number of clusters in advance prior to processing (e.g., k-mean clustering), whilst the other approach estimates the number of clusters based on the characteristics of the data. The latter can be divided into hierarchical and density-based clustering. Whilst the hierarchical method requires the researcher to determine the number of clusters based on the subjective inspection of a derived dendrogram, the density-based method estimates the cluster centres based on how data points are distributed without any user intervention. The rationale for using this technique in the current study was its good record of use for data segmentation that can recognise high-frequency groupings [29,30]. The mean-shift clustering technique used in this work is a density-based approach in which the algorithm estimates a bandwidth (BW) to merge all the data points in the vicinity of each other into a cluster (or group). The BW is based on a quantile of all the pairwise distances of the data points and affects the sensitivity as to how many groups might be recognised.

**Figure 1:**
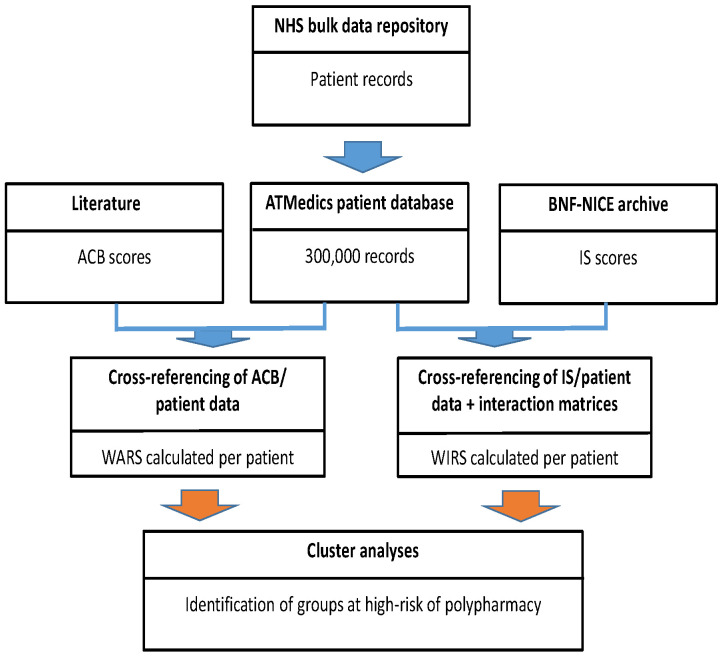
Data flow and processing. References [25,26,27].

All steps, displayed in Figure 1, were undertaken using various Python scripts developed to automate the process, prior to the vectors for the three data categories (a), (b), and (c) being presented to the mean-shift clustering algorithm. The cluster analysis itself was carried out in Python 3.7.4 using the Scikit-learn library and its default quantile value of 0.3.

### 2.4. Use Case

The process depicted in the second half of Figure 1 can be illustrated by a patient use case. A random patient record from the riskiest WARS cluster with an anticholinergic risk score of 11 was selected. The list of all medicines extracted from their prescriptions is: Betamethasone, Citalopram, Clarithromycin, Quinine, Sildenafil, Diclofenac, Omeprazole, Amitriptyline, Hydroxyzine, Promethazine, Cetirizine, and Codeine. The anticholinergic drugs from this list were extracted, their severity scores noted down, and the total score summated (Table 3).

The software also calculates the interaction risk (WIRS) from the patient’s unique interaction matrix of prescribed medicines, this being the example interaction matrix described earlier (Table 2) for which the WIRS score was 51.

This example patient was picked up by the cluster analyses based on their high WARS score and placed in the group recommended to be looked at by the healthcare professional.

## 3. Results

**Figure 2:**
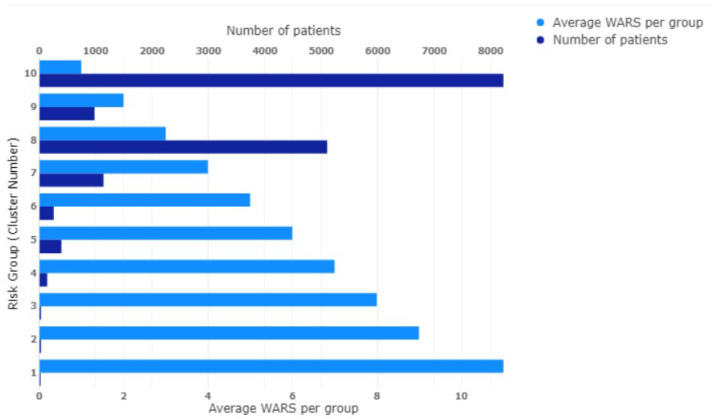
WARS patients clustered into ten different risk groups.

**Figure 3:**
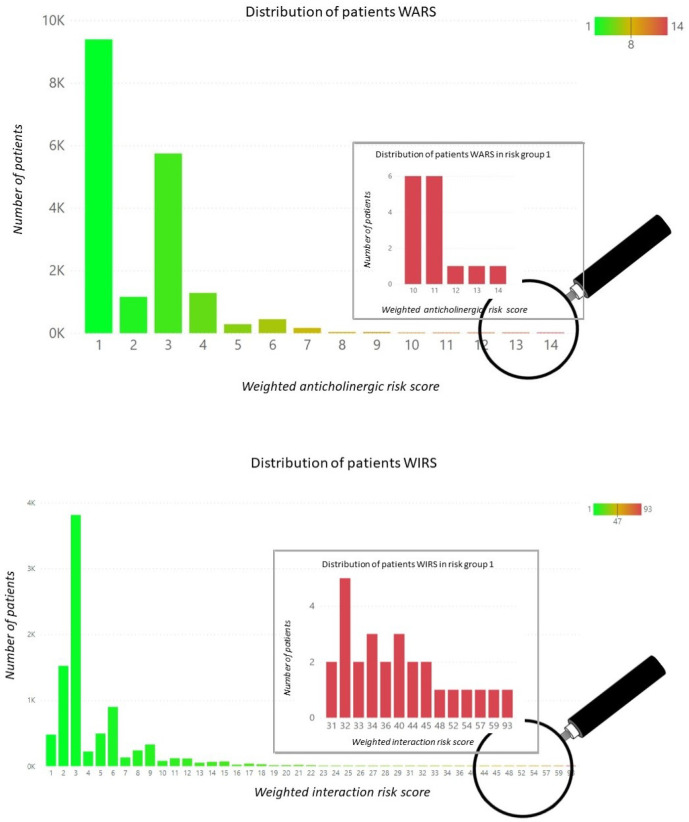
Population distributions over the calculated weighted anticholinergic risk score (**top panel**) and the calculated weighted interaction risk score (**bottom panel**). In both cases, the respective complete distributions are represented by the green-shaded histograms, whilst the embedded red histograms represent an expanded view of the respective group 1 (highest risk) distributions. The magnifying glasses of both the top and bottom panels schematically represent the approximate location of these group 1 histogram subsets within their respective population distributions.

Outcomes based on clustering applied to the WARS and the WIRS cohorts as defined by Equations (1) and (2) are reported in this section. Applying mean-shift clustering to the category (a) data (WARS data vector) returned ten clusters (i.e., ten risk groups), which are presented in Figure 2. The first cluster group of 15 patients represents the highest risk group with an average WARS of 11.00 (range is 10 to 14). The population distribution of weighted WARS values is shown in Figure 3 (top panel). For this distribution, the maximum weighted risk is 14 and the highest risk cluster group of 15 patients is shown in expanded view.

**Figure 4:**
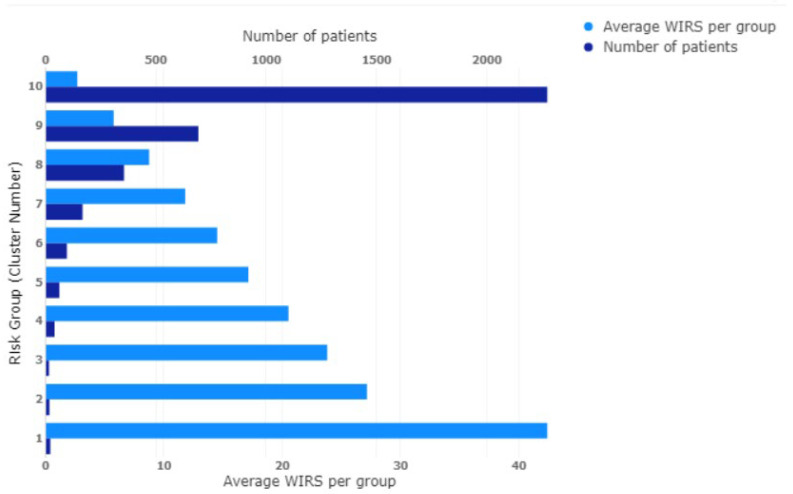
WIRS patients clustered into ten different risk groups.

Mean-shift clustering applied to the category (b) data (WIRS data vector) also groups the patients into ten risk groups with 27 patients in the first group at the highest risk with an average WIRS of 41.59 (c.f. Figure 4) with a range from 31 to 93. The corresponding population distribution of the WIRS values is shown in Figure 3 (bottom panel) and, for this distribution, the maximum weighted risk is 93 and the highest risk cluster of 27 patients is also shown in the expanded view.

**Figure 5:**
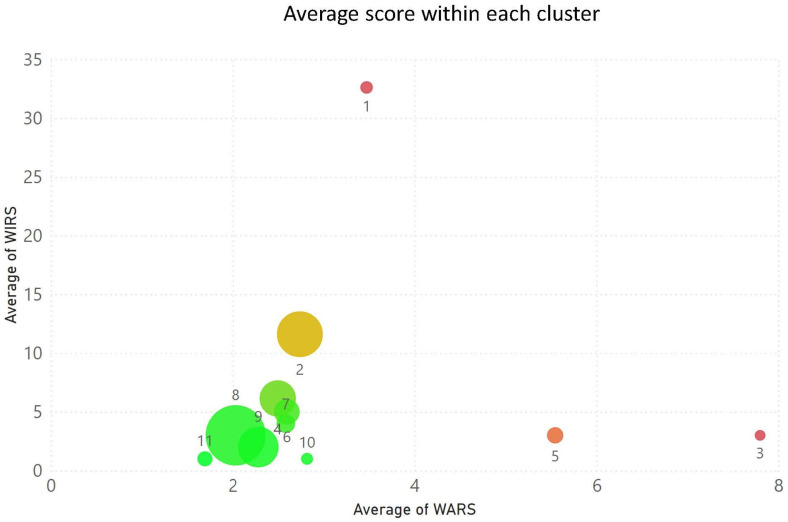
Population groups at risk from both multiple anticholinergic prescription and polypharmacy. The circle location reflects the average risk scores within each group and the circle size reflects their population. The circles are colour coded from red to amber and green in hierarchical risk order with red the highest and green the lowest risks.

Clustering that takes into account both the WARS and WIRS features was also performed to further stratify the risk. Category c) data (a two dimensional vector input of WARS and WIRS values per patient) were presented to the mean-shift clustering algorithm with the results presented in Figure 5 and Table 4. The clustering identified eleven risk groups for which some statistics are provided (tabulated data), whilst the average WIRS versus average WARS for each group were plotted in the graph and for which the radius of each group (circle) reflects that groups’ population. The groups are colour coded from red to amber and green in hierarchical risk order with red the highest and green the lowest risk, which emphasises that patients in cluster outliers 1 and 3 are at high risk (57 and 40 patients, respectively).

## 4. Discussion

Comparable works evaluating medication risk effects have used various cross-sectional studies on relatively small samples (record sizes typically in the hundreds), as in [3,4,13,14,19,31], and some also consider an AI approach [31]. Other approaches have focused on patients with specific inclusion criteria such as heart related problems and diabetes [6,31] but our technique is intended to identify patients at risk regardless of their medical condition. The majority of studies consider either anticholinergic or polypharmaceutical risk effects whilst our study evaluates them together. Our current work utilised a much larger dataset of 300,000 records using novel metrics (WARS and WIRS) and a clustering approach to group patients into different risk clusters in order to calculate new knowledge. Some studies have focused on a pharmaceutical audience utilising a number of electronic systems for managing polypharmacy [32] but, unlike our approach, none of these use machine learning algorithms that can automate the process [33]. Our conclusion that a clustering approach can successfully identify groups at high risk of polypharmacy is consistent with other approaches taken to filter big data, including records of patient medication [3,14,19].

For the data records used in our study, the significance of the single risk clustering is that a cohort of 42 patients from two groups (group 1 from WARS and group 1 from WIRS) out of 300,000 patients were identified as being at a higher risk from polypharmacy relative to the rest of this population. The automatic (and objective) recognition of this cohort presented to the healthcare professional(s) who are looking to control risks can hugely reduce their workload. Whether WARS or WIRS, it is also worth mentioning that inspection of the groups need not be limited to the first (highest) risk group.

Two important observations can be made from our results; firstly, for most of the data, the average WIRS is not correlated (or weakly correlated) with the average WARS and, secondly, the high risk outliers are high risk because they have either a high WIRS or a high WARS, but not both. These observations suggest that any systematic recognition of high risk groups should consider both polypharmaceutical and anticholinergic prescription risk measures such as WIRS and WARS, not just one of the features; otherwise, an important number of high risk patients might be missed (potentially two clusters of 97 patients in this case).

The clustering approach presented here has been embedded as a medicine safety application tool within the AT Medics population healthcare management platform. Anticholinergic and sedative medications are often used too frequently, and clinical pharmacists can significantly reduce their use using this approach. Whilst the development of our solution was based on a single time frame of the accessed patient records and risk data archives, in practice that information is constantly changing according to medical management and updates to medicinal risk data. A tool for routine primary care use needs to provide results almost instantly whilst reflecting those changes. In order to avoid unacceptable delays in real-time usage caused by communication latency, the intermediate pre-processing of records and other data can occur at pre-determined intervals (each week, for example) with only the actual clustering applied at the point of application usage (Figure 1, red arrows). This approach enables a healthcare professional to identify a subset of patients at risk (a few dozen, say) from a population database of hundreds of thousands in a few seconds—an operation that, manually, could take hours or days and be dependent on the skill and knowledge of that professional. The ability of a provider to identify and subsequently manage medication risk at a population scale markedly improves patient safety, reduces the risk of medicine-related hospital admissions and reduces unnecessary drug budget spend.

The main limitation of the present study is that patient risk was assessed based only on drug risk scores. Comorbidities, specific diagnoses, and patients’ histories were not considered, nor were environmental factors such as geography and demography. Another limitation is that the dataset only contains unlabelled raw data, so classification techniques as an alternative to clustering could not be used. Classification techniques could be considered should the data be labelled in some way.

## 5. Conclusions

One aspect of preventative health care is to use AI techniques for the identification of patients at risk from polypharmacy. Our work presents novel patient metrics of medication that reflect drug–drug polypharmaceutical risk (WIRS) and the risk between drugs of the anticholinergic drug group (WARS). These metrics were used as input to a mean-shift clustering (unsupervised learning) algorithm that grouped the data into clusters reflecting different levels of polypharmaceutical risk. Groupings based on the individual WIRS and WARS categories were returned as well as groupings based on their combined metrics. Unlike other work, this new approach was demonstrated to work with big data, processing 300,000 patient records and identifying high risk groups, each a few tens of individuals in size. This approach is safer and faster than the manual inspection of patient records, which requires up-to-date polypharmaceutical knowledge and time availability.

The clustering approach of this work has been embedded as a medicine safety application tool within the AT Medics population healthcare management platform, EZ Analytics. It has allowed the primary care team to gain unique insights into anticholinergic risk burdens across entire practice populations. The ability to easily identify high scoring clusters has meant that individuals most at risk from medicine-related harm can be prioritised for recall into planned structured medication reviews. These reviews are carried out by primary pharmacists with a focus on assessing patient understanding, adherence, and possible side effects (e.g., constipation, urinary problems, dizziness). A holistic approach is taken in partnership with the patient to jointly agree a personalised care plan. Where appropriate, this may involve de-prescribing one or more medication(s) over an agreed period with the aim of reducing the overall anticholinergic burden and its associated risks.

Future studies will incorporate other feature measures such as age, gender, and location so as to further refine the identification of high-risk patients, perhaps using hybrid AI approaches.

## Figures and Tables

**Table 1:** Some features associated with polypharmaceutical UK hospital admissions.

Medicine Measures	Risk Rate	Consequence	Treatment Duration (Months)	Age (Years)
2 or more medicines with moderate	97	Confusion	N/A	>18
or high anticholinergic activity	8	Fracture		
1 or more medicines for dementia + 1 or more medicines with moderate or high	316	Confusion	N/A	>18
anticholinergic activity	53	Fracture		
NSAID + RAS + diuretic	16	Kidney Injury	N/A	>18
Z-drug for more than one month	162	Fall	> 1	>65
	28	Fracture		
benzodiazepine for more than one month	181	Fall	> 1	>65
	32	Fracture		
benzodiazepine and Z-drug (not concurrently)	212	Fall	>1	>65
for more than one month	35	Fracture		
NSAID without gastro-protection	9	bleed	N/A	>65
NSAID + oral anticoagulant	33	bleed	N/A	>18
oral anticoagulant + anti-platelet without gastro-protection	31	bleed	N/A	>18
aspirin + anti-platelet without gastro-protection	20	bleed	N/A	>18
oral or transdermal opioid without a laxative	8	constipation	N/A	>18
oral or transdermal opioid for more than three months	18	respiratory depression, overdose poisoning or confusion	>3	>18
inhaled Long Acting Beta-agonist (LABA) without an inhaled corticosteroid (ICS)	9	exacerbation of asthma	N/A	N/A

**Table 2:** Patient interaction matrix (0 = no interaction; 3 = severe interaction).

Drugs	Amitriptyline	Betamethasone	Cetirizine	Citalopram	Clarithromycin	Codeine	Diclofenac	Hydroxyzine	Omeprazole	Promethazine	Quinine	Sildenafil
Amitriptyline	0	0	0	0	0	0	0	0	0	0	0	0
Betamethasone	0	0	0	3	3	0	3	3	0	0	3	3
Cetirizine	0	0	0	0	0	0	0	0	0	0	0	0
Citalopram	0	3	0	0	3	0	0	3	3	0	3	3
Clarithromycin	0	3	0	3	0	0	0	3	0	0	3	3
Codeine	0	0	0	0	0	0	0	0	0	0	0	0
Diclofenac	0	3	0	0	0	0	0	0	0	0	0	0
Hydroxyzine	0	3	0	3	3	0	0	0	0	0	3	3
Omeprazole	0	0	0	3	0	0	0	0	0	0	0	0
Omeprazole	0	0	0	0	0	0	0	0	0	0	0	0
Quinine	0	3	0	3	3	0	0	3	0	0	0	3
Sildenafil	0	3	0	3	3	0	0	3	0	0	3	0

**Table 3:** Anticholinergic medicines prescribed to a patient with WARS risk score 11.

Anticholinergic Name	Severity
Amitriptyline	3
Hydroxyzine	3
Promethazine	3
Cetirizine	1
Codeine	1
Total score	11

**Table 4:** WARS patients clustered into ten different risk groups.

Risk Group (Cluster Number)	Number of Patients	Average Risk per Group
1	15	11.00
2	36	9.00
3	37	8.00
4	167	7.00
5	446	6.00
6	287	5.00
7	1286	4.00
8	5744	3.00
9	1161	2.00
10	9389	1.00

## Data Availability

Patient data records used for this study are not available due to data protection constraints. Anticholinergic risk and drug–drug risk data are freely available from the sources listed in this paper. An example subset of anonymised metadata and derived data used for the clustering analysis is available from the corresponding author.

## Abbreviations

The following abbreviations are used in this manuscript:

WIRS	Weighted Interaction Risk Score
WARS	Weighted Anticholinergic Risk Score
AI	Artificial Intelligence
ADRs	Adverse Drug Reactions
ACB	Anticholinergic Cognitive Burden scale
BNF-NICE	British National Formulary—National Institute for Health and Care Excellence
IS	Interaction Severity
BW	bandwidth
